# Single center experience with a novel single-branched thoracic stent graft

**DOI:** 10.1186/s42155-025-00545-y

**Published:** 2025-07-08

**Authors:** Theodoros Kratimenos, Dimitra Tachmetzidi Papoutsi, Panagiotis Petaloudis, Nefeli Ntinou, Myrto Papadopoulou, Vasileios Panou, Evaggelia Kalaitzidou, Dimitrios Tomais, Ilias Samiotis, Loukia Alexopoulou-Prounia, Panagiotis Dedeilias, Mihalis Argiriou

**Affiliations:** 1 Department of Interventional Radiology, General Hospital of Athens Evangelismos, Athens, Greece; 2 Department of Cardiothoracic Surgery, General Hospital of Athens Evangelismos, Athens, Greece

**Keywords:** TEVAR, Branched stent grafts, LSA

## Abstract

**Background:**

Thoracic endovascular aortic repair (TEVAR) has been a feasible treatment option since the first stent graft was approved by the FDA in 2005, and is now the recommended method for treating most descending aorta pathology in the current clinical practice guidelines. Indications for TEVAR include descending aorta aneurysms, traumatic aortic injury and pathology that presents as acute aortic syndrome. More often than not the lesion that needs to be excluded is quite close or contains the distal aortic arch, thus requiring the coverage of the left subclavian artery (LSA) origin, a practice that has been associated with severe complications. Contraindications to LSA coverage resulted in the development of various surgical and endovascular LSA revascularization techniques.

**Materials and methods:**

Branched stent grafts containing a single branch for the LSA are a rapidly evolving technology regarding LSA reconstruction during TEVAR. The aim of this article is to demonstrate our center’s experience using a novel off-the-shelf single-branched stent graft, the GORE® TAG® Thoracic Branch Endoprosthesis (TBE) (W. L. Gore & Associates, Inc, Flagstaff, Ariz, USA). The GORE® TAG® TBE is commercially available in Europe since early 2024. We have so far, since February 2024, treated 12 patients using this endograft, successfully treating all types of aortic lesions.

**Conclusions:**

Branched TEVAR is becoming a feasible option for treating descending aorta pathology, without covering the LSA. Moreover, the development of off-the-shelf branched stent grafts, enables physicians to treat patients in the emergency setting, aside from planned procedures.

## Background

**Fig. 1:**
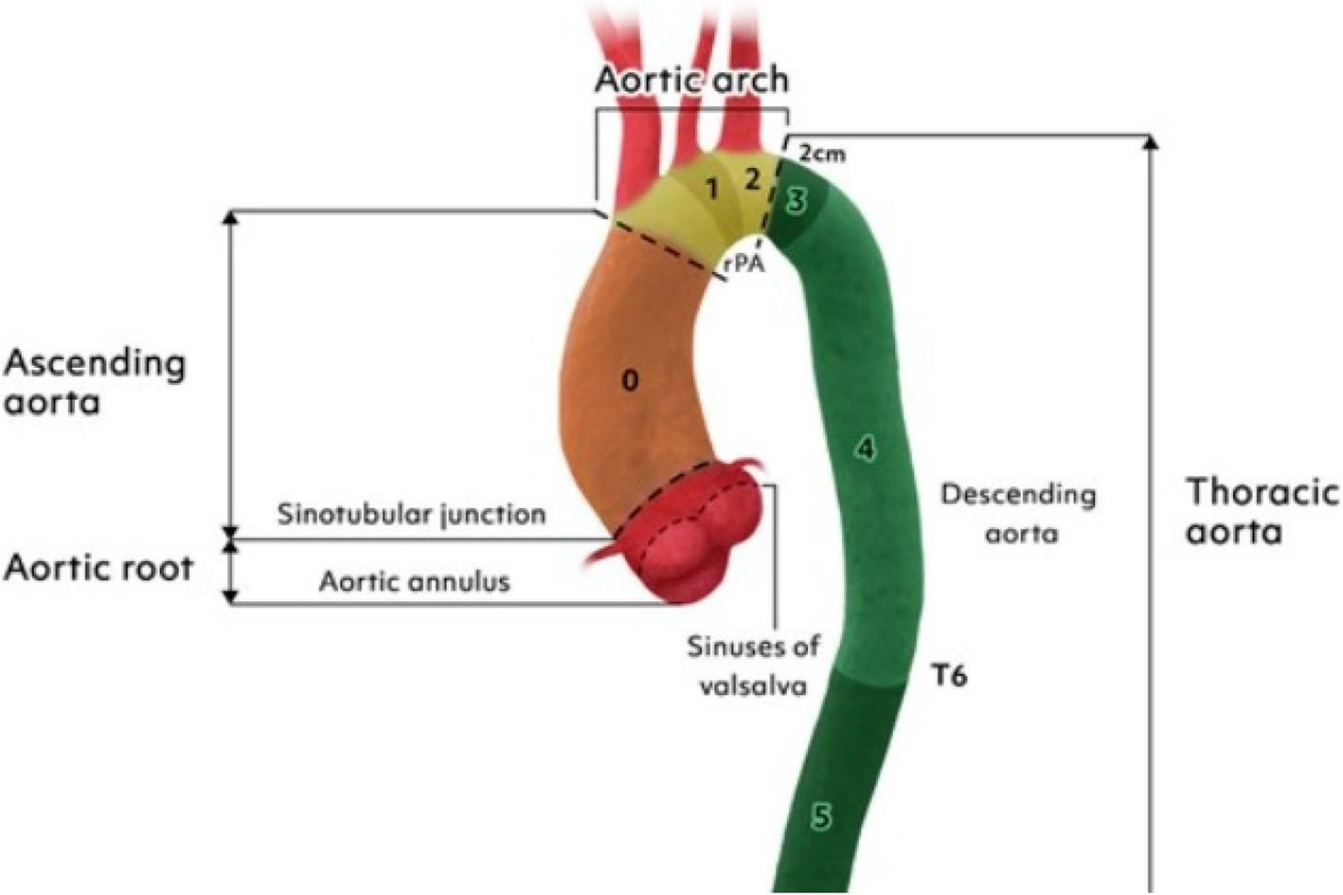
The Ishimaru aortic zones [2]

Descending thoracic aorta disease has traditionally been treated surgically up until the first endograft was approved by the U.S. Food and Drug Adminstration in 2005 [1]. Since then, thoracic endovascular aortic repair (TEVAR) has progressed from being a feasible treatment option for patients deemed unsuitable for open surgery, to being the first line intervention for most descending aorta pathologies in recent guidelines [1–3]. Due to the high incidence of proximal descending thoracic aorta lesions, LSA coverage is often required during TEVAR (range 10% to 50%) in order to achieve an adequate proximal seal of the stent graft [4]. The relatively rare but devastating complications arising from LSA coverage and the cases in which it is contraindicated, led to the development of indications for revascularization of the LSA during TEVAR [4]. Although hybrid procedures including surgical revascularization and TEVAR remain the standard of care, several totally endovascular procedures for LSA revascularization have emerged [1–5]. Among the latest advancements regarding TEVAR with an Ishimaru zone 2 (Fig. 1) landing zone is the development of single-branched thoracic stent grafts. In the following article we aim to present our experience and short term results, using the recently commercially available in Europe GORE® TAG® Thoracic Branch Endoprosthesis (TBE).

## Materials and methods

### Patient population

**Fig. 2:**
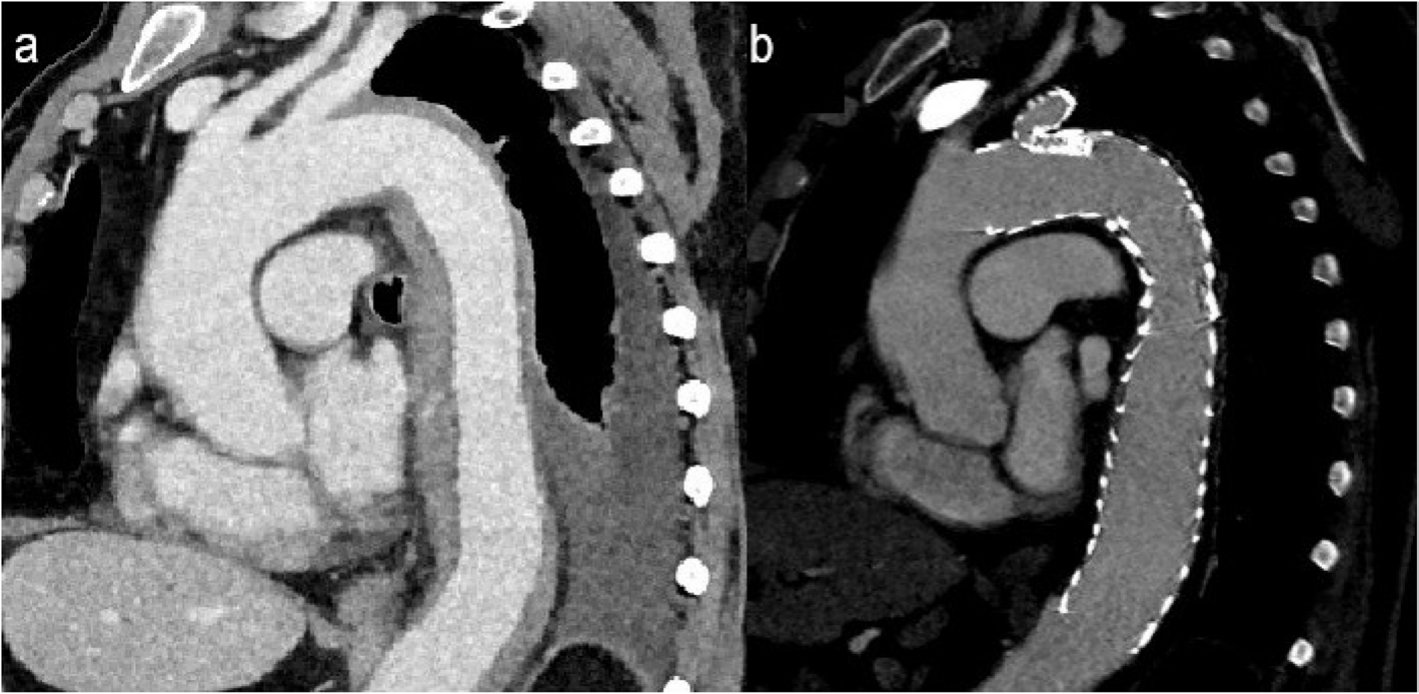
Saggital CTA reconstructed images of a patient with an intramural hematoma before (**a**) and after (**b**) treatment with the single-branched stent graft

**Fig. 3:**
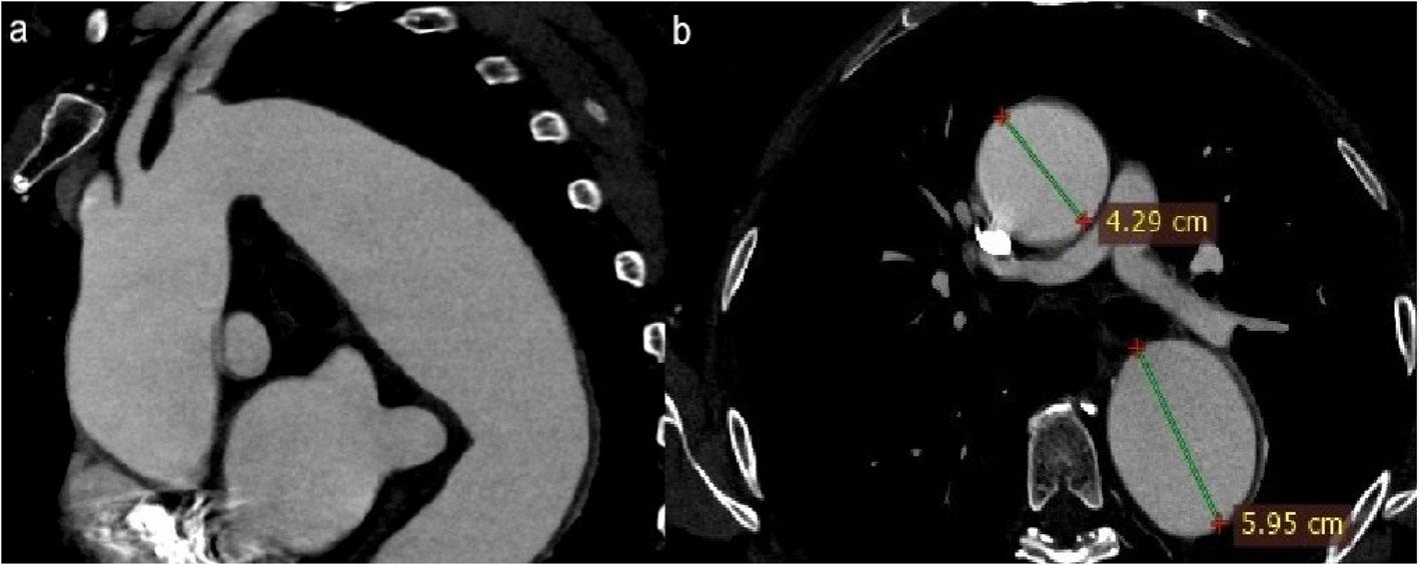
Saggital reconstruction and axial CTA image shows a large descending aorta aneurysm originating very close to the LSA ostium

**Fig. 4:**
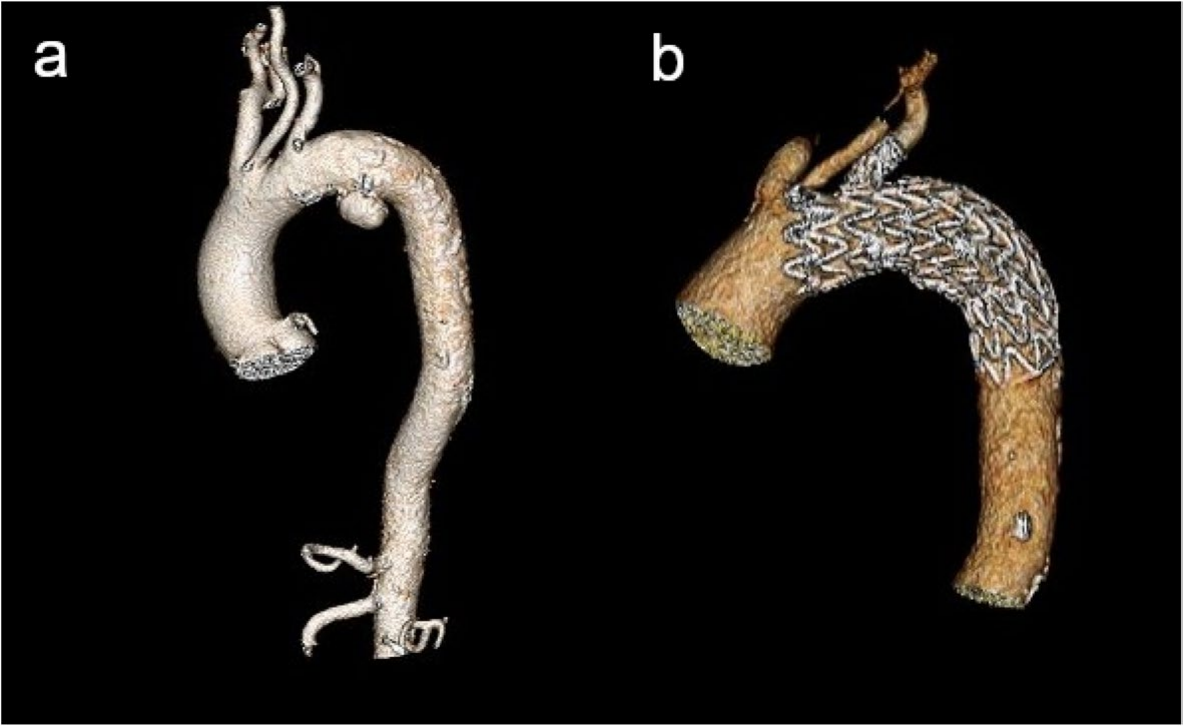
3D reconstructed CTA images of a patient with a descending aorta aneurysm before (**a**) and after (**b**) treatment with the single-branched stent graft

From February 2024 to December 2024 twelve patients with descending aorta disease have been treated at our institution receiving a single-branched thoracic endoprosthesis. All the patients displayed aortic pathology which emerged very close to the distal part of the LSA ostium and needed a zone 2 landing zone TEVAR (Figs. 2, 3, 4). Their demographic data and indication for intervention are displayed in Table 1.

**Table 1:** Demographic data and indication for intervention of the patients treated at our institution (*TBAD *Type B aortic dissection, *DTAA *Descending thoracic aorta aneurysm, *TAI *traumatic aortic injury, *IMH *intramural hematoma, *PAU* penetrating aortic ulcer)

Variable	Mean age (years)	Male sex	Hypertension	Tobacco use	Coronary heart disease
Patients (*n* = 12)	61,75	10	7	9	4
TBAD (*n* = 5)	61,4	5	2	3	0
DTAA (*n* = 3)	66,3	2	2	2	1
TAI (*n* = 1)	33	1	0	1	0
IMH (*n* = 2)	73,5	1	2	2	2
PAU (*n* = 1)	55	1	1	0	1

### Branched endoprosthesis

All patients were treated using the GORE® TAG® TBE (W. L. Gore & Associates, Inc, Flagstaff, Ariz, USA). The branched stent graft, as described in the enclosed instructions for use document, consists of a main aortic stent graft, the aortic component (AC), a side branch component for the LSA (SB) and an optional aortic extender (AE), which may be used to improve sealing of the AC and/or extend sealing zone proximally. The AC incorporates an internal portal, measuring 8 mm in diameter, that opens to the outer device surface. The SB component is inserted through this internal portal into the LSA using a second guidewire, thus allowing blood flow to the LSA. All the aforementioned components consist of nitinol stents and expanded polytetrafluoroethylene (ePTFE) graft material. Gold radiopaque markers are placed on either end of the AC, on the internal portal of the AC and on either end of the SB component. The leading end of the AC bears partially uncovered stent apices. Proximal landing zone between the distal part of the LCCA ostium and the proximal part of the LSA ostium must be at least 2 cm. The device also comes with a 12 mm internal portal which may be used in hybrid procedures with a zone 0 proximal landing zone, requiring a 4 cm proximal landing zone. Distal landing zone must be at least 2 cm proximal to the celiac artery. The device may be used for intended aortic diameter of 16 mm to 42 mm and intended LSA diameter of 6 mm to 18 mm, measuring intima to intima, and oversizing is incorporated in the sizing tables found in the instructions for use document. LSA minimum length, measured along the outer curvature of the LSA from the ostium to the first major branch vessel, should be between 2,5–3 cm. An adequate distal access site is required as the device is designed to be introduced through a 20F to 26F sheath.

### Procedure

**Fig. 5:**
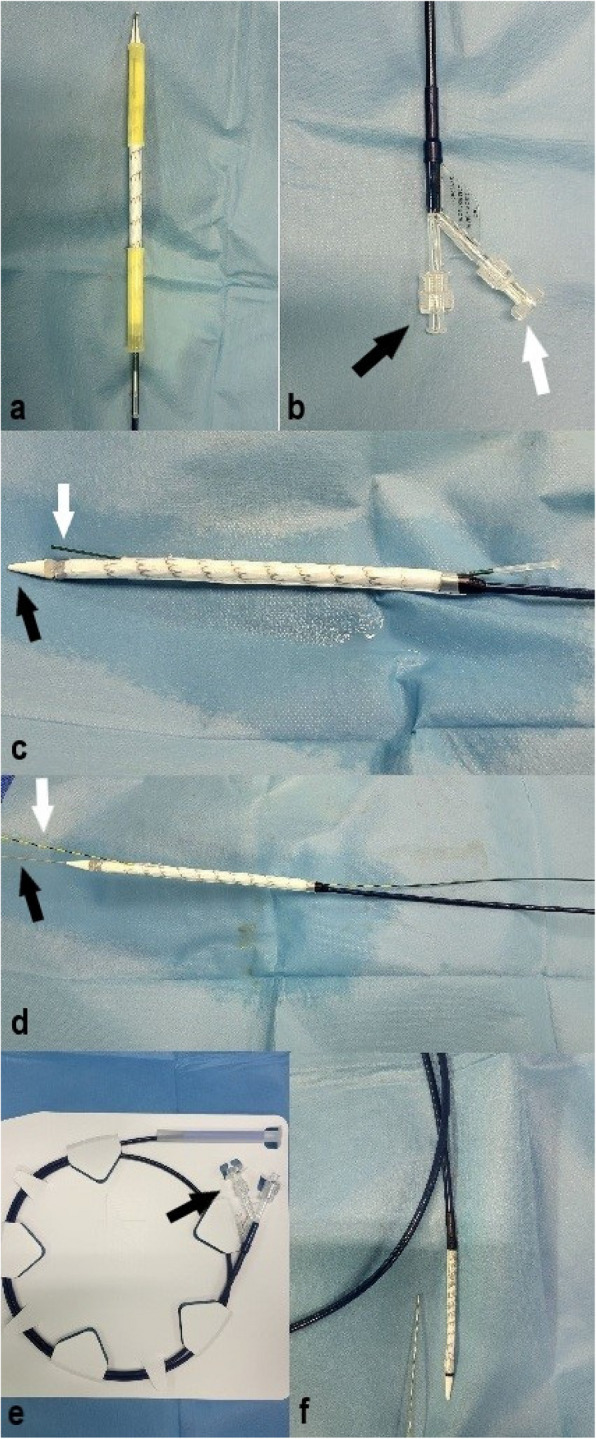
The Gore TBE device. **a** The AC; the proximal end of the device after it is taken out of its package. **b** The distal end of the AC device; the black arrow points at the point of insertion of the super stiff guidewire and the white arrow shows the deployment knob of the AC. **c** The device before it is loaded on both guidewires; the black arrow points at the olive tip and white arrow shows the removable guidewire tube which is how the pre-cannulation of the AC’s internal portal is achieved. **d** The AC after it is loaded on both guidewires, the extra stiff (black arrow) and the stiff through-and-through guidewire (white arrow). **e** The SB component of the device; arrow shows its deployment knob. **f** The SB component before it is loaded onto the through-and-through guidewire

**Fig. 6:**
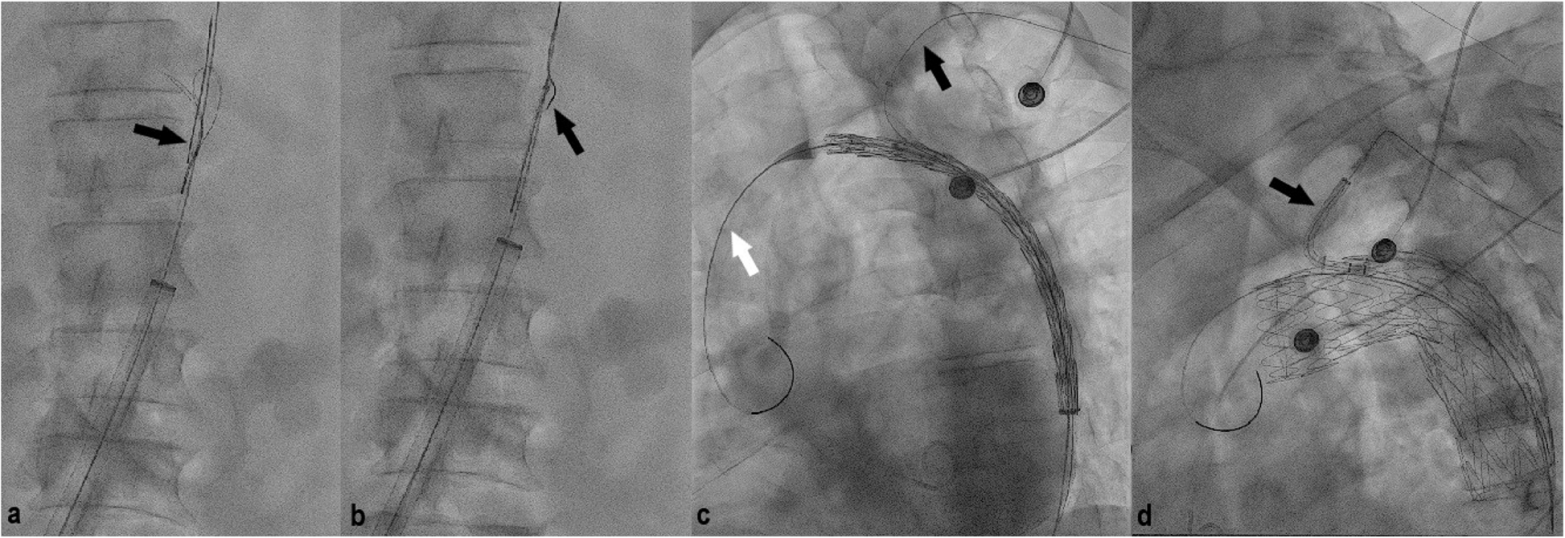
Digital subtraction angiography images throughout the procedure regarding different patients. **a**, **b** The through-and-through guidewire is snared in the descending aorta; black arrow points at the tip of the through-and through guidewire. **c** The stent graft is advanced into position avoiding entanglement between the two guidewires; the black arrow points at the through-and-through guidewire. **d** The SB component is advanced into position, through the stent graft’s internal portal

**Fig. 7:**
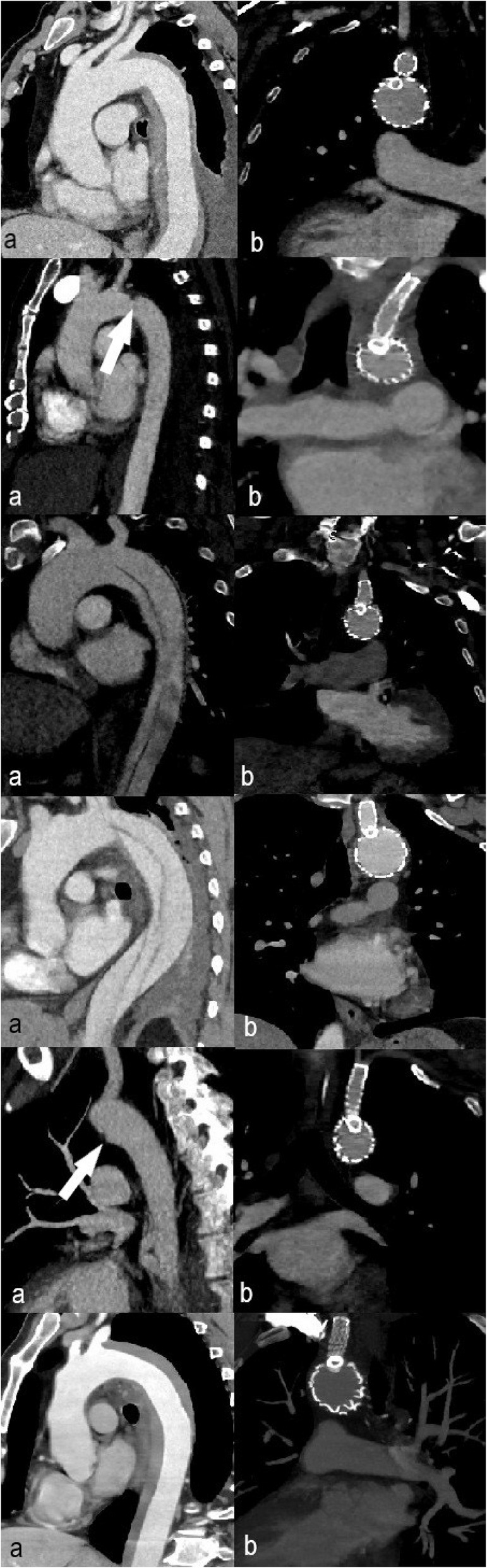
**a** Pre-procedural reconstructed CTA images and (**b**) 6-months post-procedural reconstructed CTA images. Each row represents an individual patient treated in our center. 1st row: IMH, 2nd row: PAU (white arrow, a), 3rd row: TBAD, 4th row: TBAD, 5th row: TAI (white arrow shows traumatic pseudoaneurysm, a), 6th row: IMH

Pre-operative Computed Tomography angiographic (CTA) scans (slice thickness 0.5-2 mm) were obtained in all the treated patients and the appropriate device size was selected for each patient. Patients were placed in supine position. Both inguinal regions and left arm were surgically prepared and draped. Conscious sedation, prophylactic antibiotics and bolus heparin (5.000 IU) were administered perioperatively. The distal access site used for device delivery was the common femoral artery, which was exposed surgically and then cannulated with a 6F sheath. The contra-lateral femoral artery was percutaneously punctured for diagnostic aortography. In three patients diagnostic aortography was obtained through a right brachial artery site, due to the extension of the dissection to the contralateral common femoral artery. A super-stiff guidewire (Lunderquist® Extra-Stiff Wire Guide, Cook, Bloomington, IN, USA) was advanced into the ascending aorta through ipsilateral access site. Catheterization of the descending aorta from the brachial access site and advancement of the second stiff, 0,035″ guidewire (Jagwire™ High Performance Guidewire, Boston Scientific, Massachusetts, USA) into the descending aorta was then performed. The 6F sheath on the distal access site was then exchanged with the 20-24F sheath, depending on the selected device size (GORE® DrySeal Flex Introducer Sheath, W. L. Gore & Associates, Inc, Flagstaff, Ariz, USA). Snaring of the second guidewire was performed through the femoral access site in the descending aorta, achieving a through-and-through wire access. Aortogram images were then obtained and the C-arm was properly positioned for accurate procedural fluoroscopic imaging. Before introducing it into the femoral sheath, the stent grafts’ delivery system is loaded on both guidewires, the super stiff guidewire and the secondary through-and-through guidewire. The secondary through-and-through guidewire is inserted into the removable guidewire tube which is attached to the delivery system, thus achieving the pre-cannulation of the internal portal of the AC (Fig. 5c). Under fluoroscopic guidance, the delivery catheter was advanced to the proximal descending aorta, cautiously avoiding twists between the two guidewires (Fig. 6a-c). After correct positioning of the delivery sheath was achieved, the stent graft was deployed by pulling the deployment knob. Deployment initiates from the portal opening and extends simultaneously to the proximal and distal ends of the endoprosthesis. The side branch component was then loaded onto the secondary through-and-through guidewire and advanced into position, with its proximal part in the LSA and the distal part in the internal portal of the AC (Fig. 6d). Deployment of the SB component is also attained by pulling the deployment knob. The Aortic Extender was used in one case. Ballooning of the main endoprosthesis in the cases indicated and side branch ballooning throughout its length were then performed. Final aortogram was obtained without signs of endoleaks in any of the patients. Brachial access site and contralateral femoral site were sealed using manual compression and the femoral access site was surgically closed. All patients receive double antiplatelet therapy post-operatively for six months and then single antiplatelet therapy for life. Ten out of twelve patients proceeded to 1 month follow up, out of which only one displayed a type II endoleak from a brochial artery, without signs of aneurysmal sack enlargement. So far, seven of our patients have undergone a CTA for their 6-month follow up, displaying no signs of type I or type III endoleaks, stent migration or stent fracture (Fig. 7). Patients receiving thoracic stent-grafts should undergo a CTA at 6 and 12 months post-operatively and then yearly thereafter [2]. Procedural and follow-up details are shown on Table 2.

**Table 2:** indication for intervention, procedural and follow up data for each of our patients (*TBAD *Type B aortic dissection, *DTAA *Descending thoracic aorta aneurysm, *TAI *traumatic aortic injury, *IMH *intramural hematoma, *PAU *penetrating aortic ulcer, *NA *not available)

	**Aortic pathology**	**Flouroscopy time (minutes)**	**Technical success**	**Hospital stay (days)**	**30 day survival**	**6 months FU**
Patient 1	IMH	12,6	100%	5	Yes	no complications
Patient 2	TBAD	16,2	100%	3	Yes	no complications
Patient 3	ΤΑΙ	15,5	100%	48	Yes	no complications
Patient 4	DTAA	11,7	100%	2	Yes	type II endoleak, no enlargement of aneurysmal sack
Patient 5	TBAD	16,6	100%	3	Yes	no complications
Patient 6	PAU	8,6	100%	10	Yes	no complications
Patient 7	IMH	13	100%	2	Yes	no complications
Patient 8	DTAA	16,1	100%	3	Yes	NA
Patient 9	TBAD	19,9	100%	2	Yes	NA
Patient 10	TBAD	26,4	100%	5	Yes	NA
Patient 11	DTAA	12,9	100%	15	Yes	NA
Patient 12	TBAD	17,5	100%	18	Yes	NA
Mean		**15,58**	**100%**	**9,7**		

## Discussion

TEVAR is currently the recommended treatment for descending thoracic aorta disease, as it is associated with lower mortality and morbidity rates than open surgery [1–3]. As a method it was initially indicated only for aortic aneurysmal disease, but following the rapid advancement of endovascular therapies, its indications have expanded to include traumatic aortic injury and acute aortic syndrome pathology (type B aortic dissection, intramural hematoma, penetrating aortic ulcers). Endovascular repair is the first line intervention in these patients, either in the emergent setting or electively according to the most recent guidelines [2, 3]. Contraindications to TEVAR include an unfavorable patient anatomy and an infected placement field [1]. Anatomic features that need to be considered preoperatively are extreme tortuosity, distal vascular access and adequate proximal and distal sealing zones [1]. Failure to achieve a secure seal at either end of the endograft may result in a type I endoleak, device migration or bird-beak configuration of the endograft [4]. Current research and development in graft technology aim to extend endovascular repair to the aortic arch using various techniques, such as chimney TEVAR, fenestrated TEVAR and custom made branched endografts, thus avoiding open surgery higher mortality and complications [2, 6]. Nonetheless, the technical difficulties and complications of chimney and fenestrated TEVAR and the anatomic limitations and long manufacturing hours of custom made branched endografts, often pose a challenge and exclude the endovascular repair from the available options for total arch repair [2, 6]. This however is not the case when treating descending aorta lesions with a zone 2 landing zone TEVAR.

Studies have shown that coverage of the LSA during TEVAR is often required (rates range between 10 and 50%), in order to achieve an adequate proximal endograft landing zone [4]. In trauma patients with traumatic aortic injury, 30% require LSA coverage during TEVAR [3]. Most patients undergoing TEVAR with LSA coverage recover well, without the need for further interventions [1, 4]. In these patients a reversal in blood flow in the left vertebral artery, supplied by the circle of Willis, provides blood flow in the LSA [4]. The aforementioned collateral pathway however is not always the case and LSA coverage can lead to devastating complications, such as stroke, spinal cord ischemia, left upper extremity ischemia and myocardial infarction [4]. These complications and the ongoing graft technology advancement have resulted in the 2024 European Association for Cardio-Thoracic Surgery/ Society of Thoracic Surgeons guidelines, where LSA revascularization is upgraded to a strong recommendation in any zone 2 landing zone TEVAR [2].

The hybrid technique, which consists of TEVAR and surgical revascularization of the LSA, has been the standard method of treatment [5–7]. Although it is a well-established method for LSA revascularization, it is not without complications, which include those regarding the surgical trauma and various local nerve injuries [4, 5]. Total endovascular techniques for LSA revascularization reduce operative time and complications [5, 6]. The chimney technique, in vitro and in situ fenestration and branched stent grafts are the endovascular alternatives to surgical LSA reconstruction when performing TEVAR with a zone 2 landing zone. The chimney technique for LSA revascularization has proven quite useful in bail-out situations, but has been associated with type Ia endoleaks originating from the gutter between the two stent grafts [6, 7]. Moreover, it is prone to branch stent graft occlusion resulting from the constant pressure of the aortic stent graft onto the branch [6, 7]. In in vitro and in situ fenestration the aortic stent graft is fenestrated, either on the table or inside the patient, and placed with its fenestrations in alignment with the branch ostium. Both fenestrating techniques are relatively difficult and time- consuming and can result in endoleaks due to graft tears, while the in situ fenestration technique has also been associated with cerebral ischemia because of the temporary coverage of aortic arch branches [5, 6].

Branched stent-grafts for treating aortic arch pathology are one of the most recent developments in graft technology. Custom made multi branched devices now allow TEVAR extension up to zone 0. Branched endografts are associated with lower rates of endoleaks and perioperative neurological adverse events than the aforementioned techniques, while demonstrating better fixation on the aortic curvature [7]. Single branch stent grafts for zone 2 landing zone incorporate a branch stent graft for the LSA. Such a stent graft is the GORE® TAG® TBE, an off-the-shelf device with promising results so far, both in emergent and elective setting [8, 9]. Procedural mortality and hospital stay in patients treated with this endograft were similar to those treated with the hybrid technique, according to early trial results [8, 9]. Early adverse events and procedural stroke rate were also comparable to those of standard TEVAR [7]. Regarding our experience with the GORE® TAG® TBE, stent deployment and side branch patency were 100% successful perioperatively and procedure time shortened as we became more familiar with the device. The aortic pathology was excluded from systematic circulation and LSA blood flow was maintained in all patients.

## Conclusion

Treating aortic pathology has always posed a challenge for physicians. Advances in endovascular procedures and graft technology have expanded the numbers of patients eligible for treatment and have drastically lowered mortality and morbidity rates compared to open surgery. The development of a single-branch endoprosthesis for zone 2 landing zone TEVAR, it being an off the shelf device nonetheless, allows physicians to treat patients in the emergent setting as well as electively, safely and effectively, without the need of surgical LSA reconstruction. From our experience, serving in a trauma center, this is of utter importance considering that trauma patients often display aortic injuries requiring zone 2 landing zone TEVAR.

## Data Availability

Data sharing is not applicable to this article as no datasets were generated or analysed during the current study.

## Abbreviations

TEVAR: Thoracic Endovascular Aortic Repair
LSA: Left Subclavian Artery
LCCA: Left Common Carotid Artery
TBE: Thoracic Branched Endoprosthesis
AC: Aortic Component
SB: Side Branch
AE: Aortic extender
CTA: Computed Tomography Angiography
TBAD: Type B aortic dissection
DTAA: Descending thoracic aorta aneurysm
TAI: Traumatic aortic injury
IMH: Intramural hematoma
PAU: Penetrating aortic ulcer
